# The Self-Reported Quality of Sleep and Its Relationship with the Development of Arterial Hypertension: Perspectives from the Tlalpan 2020 Cohort

**DOI:** 10.3390/jcm13206089

**Published:** 2024-10-12

**Authors:** Luis M. Amezcua-Guerra, Kelly P. Velázquez-Espinosa, Lizbeth A. Piña-Soto, Guadalupe O. Gutiérrez-Esparza, Mireya Martínez-García, Malinalli Brianza-Padilla

**Affiliations:** 1 Department of Immunology, Instituto Nacional de Cardiología Ignacio Chávez, Tlapan, Mexico City 14080, Mexico; lmamezcuag@gmail.com (L.M.A.-G.); kpve03@gmail.com (K.P.V.-E.); gues.02@gmail.com (G.O.G.-E.); mireya.martinez@cardiologia.org.mx (M.M.-G.); 2 Health Care Department, Universidad Autónoma Metropolitana-Xochimilco, Coyoacán, Mexico City 04960, Mexico; 3 Red MEDICI, Carrera de Médico Cirujano, Facultad de Estudios Superiores Iztacala, UNAM, Tlalnepantla de Baz 54090, Mexico; lizbethpina@gmail.com; 4 Researcher for Mexico CONAHCYT, Consejo Nacional de Humanidades, Ciencias y Tecnologías, Mexico City 08400, Mexico

**Keywords:** sleep, sleep quality, wakefulness, sleep-disordered breathing, hypertension, risk factors

## Abstract

**Background/Objectives:** A well-established association exists between the development of hypertension and sleep quality. The connection between self-reported sleep quality and the onset of hypertension is particularly significant in populations with metabolic deterioration, such as in Mexico. **Methods:** The Tlalpan 2020 Cohort was analyzed to explore this association. Clinical and anthropometric characteristics, along with the Medical Outcomes Study Sleep Scale (MOS-SS), were compared between participants who developed hypertension and those who did not over a follow-up period of 30.8 months. The potential role of poor sleep quality in the development of hypertension was assessed. **Results:** Among 1520 participants, 12% developed hypertension. These individuals had higher anthropometric and laboratory values and reported poorer sleep quality. An elevated sleep problems index was associated with a 50% higher relative risk of developing hypertension (OR: 1.5; 95% CI: 1.087 to 2.069). Additionally, self-reported snoring was associated with hypertension onset (36.3 vs. 43.3; *p* = 0.019). **Conclusions:** Poor sleep quality and respiratory disturbances during sleep increase the risk of developing hypertension. Furthermore, hypertension was associated with snoring, highlighting the importance of early interventions to improve sleep quality.

## 1. Introduction

Arterial hypertension is a chronic condition widely recognized as a significant risk factor for cardiovascular diseases. The global prevalence of hypertension has doubled from 1990 to 2019, yet less than half of the affected individuals achieve adequate disease control [1]. Middle-income countries bear a disproportionate burden of hypertension, coupled with considerable population aging [2]. In Mexico, the prevalence of hypertension in adults is 47.8% (according to the American College of Cardiology/American Heart Association (ACC/AHA) criteria) [3]. Of these, 65.5% are unaware of their diagnosis. Among adults with a previous diagnosis of hypertension, 33.7% have controlled blood pressure. According to the Eighth Joint National Committee (JNC-8) classification, 29.4% of adults have hypertension, and 43.9% are unaware of their diagnosis [4].

Hypertension is a multifactorial disease, with sleep disturbances being a well-recognized risk factor [1]. Sleep is a complex biological process crucial for the restoration of physical, metabolic, and physiological functions, including blood pressure regulation [1]. Like the sleep–wake cycle, blood pressure exhibits an endogenous rhythm of nearly 24 h. The synchronization of neuronal and hormonal outputs, governed by the suprachiasmatic nucleus (SCN), facilitates appropriate blood pressure responses. Anatomical alterations in the SCN of hypertensive patients suggest that circadian disruptions or desynchronizations may contribute to cardiovascular dysfunction [5].

Data on sleep quality in Mexico are outdated. One major obstacle is the limited access to polysomnography equipment due to the infrastructure and technical complexity required for sleep studies [6]. To address this, various clinimetric instruments have been developed to indirectly evaluate sleep quality, showing good correlation with findings from advanced electrophysiological studies, including polysomnography [7]. It is known that living in urban areas and being over 40 years of age increases the risk of insomnia, shorter sleep duration, and obstructive sleep apnea (OSA). These conditions are concerning due to their strong association with increased hypertension, glucose metabolism disorders, and accident risk [8].

Current evidence suggests an association between poor subjective sleep quality and the development of hypertension [9]. Despite variations in data acquisition methods, social perceptions, and socio-environmental factors, subjective sleep assessments remain revealing and reliable. This study aims to analyze the efficacy of a self-reported clinimetric instrument in identifying major sleep problems and predicting the future development of hypertension in a cohort of middle-aged adults in Mexico City.

## 2. Materials and Methods

### 2.1. Study Design

This study analyzed data from the Tlalpan 2020 Cohort, a longitudinal investigation into the incidence of hypertension. The study included 1520 clinically healthy adults, aged 20–50 years, residing in Mexico City. Participants were recruited between 2014 and 2019, with biennial follow-ups. Pregnant or breastfeeding women and individuals with cardiovascular disease, cancer, cognitive and mental disabilities, chronic infections, or inflammatory and autoimmune disorders were excluded. Additionally, participants with elevated blood pressure or fasting glucose levels during the initial visit and those who did not provide complete information [10] were excluded.

Data collection and management were conducted using an electronic case report form.

### 2.2. Data Collection

Data collection during initial visit and follow-up visits included clinical, anthropometric, biochemical, and sleep quality information. Clinical and anthropometric measurements such as body mass index (BMI), waist circumference, height, and weight were obtained through individual interviews following the guidelines of the International Society for the Advancement of Kinanthropometry [11].

### 2.3. Laboratory Data

Laboratory analyses were performed on blood samples obtained after a 12 h overnight fast. These analyses included measurements of uric acid, serum creatinine, total cholesterol, high-density lipoproteins (HDL-C), low-density lipoproteins (LDL-C), triglycerides, glucose, iron, plasma, and urine sodium and were conducted at the core lab of our hospital.

### 2.4. The Medical Outcomes Study Sleep Scale (MOS-SS)

The main sleep problems were assessed using the Spanish version of the Medical Outcomes Study Sleep Scale (MOS-SS), a patient-reported, non-disease-specific instrument for evaluating sleep outcomes. The scale was designed as a 12-question self-report questionnaire that addresses six domains related to sleep problems:

Sleep disturbance (SLPD4): Questions 1, 3, 7, and 8; related to the time it takes for the person to fall asleep and how they perceive the quality of their sleep.
Snoring (SLPSNR1): Question 10; focused on the perception of snoring.
Sleep awakening short of breath or with headache (SLPSOB): Question 5; a specific inquiry about these symptoms upon waking.
Sleep adequacy (SLPA): Questions 4 and 12; focused on whether the person feels they sleep enough and feel rested.
Somnolence (SLPS): Questions 6, 9, and 1; focused on describing whether the person feels tired or sleepy during the day.
Quantity of sleep (SLPQ): Question 2; a specific question about the number of hours of sleep.

Additionally, the MOS-SS scale generates the SLP9 sleep problems index from 9 of the 12 items (1, 3, 4, 5, 6, 7, 8, 9, and 12) and its shorter version, the SLP6 index, from 6 items (4, 5, 7, 8, 9, and 12) [12].

Most of the questionnaire responses were recorded on a Likert scale, considering experiences over the past four weeks, and recoded to a 0–100 range. In this format, higher scores on subscales (SLPD4, SLPSNR1, SLPSOB, and SLPS) and indices (SLP6 and SLP9) indicate greater sleep problems, while lower scores on subscales (SLPA and SLPQ) indicate significant sleep problems [13]. The description of the data can be found in the Supplementary Materials.

### 2.5. Measurement of Blood Pressure

Blood pressure was measured three times on the left arm at 3 min intervals after the participant had been seated for at least 10 min. If one of the three measurements was considerably different, an additional measurement was taken. The reported blood pressure was the average of the three measurements, using an appropriately sized cuff and a mercury sphygmomanometer calibrated at our hospital. Hypertension was defined as a systolic blood pressure ≥ 140 mm Hg and diastolic blood pressure ≥ 90 mm Hg [14].

### 2.6. Statistical Analysis

Statistical analysis was performed using data from both the initial and follow-up visits of the Tlalpan 2020 Cohort. The Kolmogorov–Smirnov test was used to assess population distribution. Qualitative variables were reported as percentages, and quantitative variables were expressed as mean ± standard deviation. Due to non-parametric distribution, Fisher’s exact test and the Mann–Whitney test were used to assess differences between groups. The statistical analysis was conducted with 80% power and a 95% confidence level.

Two primary analyses were conducted as follows.

#### 2.6.1. Cross-Sectional Analysis of the Initial Visit

The analysis included 1520 participants who had complete data from the MOS-SS questionnaire at baseline and maintained follow-up for at least 2 years. Participants were categorized based on whether they developed hypertension during the study period. Fisher’s exact tests and the Baptista–Pike odds ratios were used to assess the relationship between the SLP9 index and development of hypertension. A multiple logistic regression adjusted for age, sex, body mass index, lipids, glucose, and systolic and diastolic blood pressure was performed to avoid confounding variables. Although a healthy population was studied, SBP and DBP values may have been higher at baseline and could be predictors of the development of hypertension. Hypertension was taken as the dependent variable to determinate the risk conferred by the MOS-SS scale domains and indices. A receiver operating characteristic (ROC) curve was constructed using the Youden index to identify the optimal cut-off point of SLP9 for predicting hypertension development. Additionally, a survival curve analysis and hazard ratio calculation were performed to evaluate the risk over time.

#### 2.6.2. Follow-Up Visit (MOS-SS before and after Developing Hypertension)

A sub-analysis was conducted including 63 participants who developed hypertension during the follow-up period and had completed MOS-SS questionnaires at both baseline and post-hypertension diagnosis. The Wilcoxon signed-rank test was used to assess differences between the baseline and follow-up assessments.

Statistical significance was set at *p* ≤ 0.05. All data analyses and graph designs were performed using GraphPad Prism v.10 software (GraphPad Inc.; La Jolla, CA, USA).

## 3. Results

### 3.1. Initial Visit

The analyses in our study were based on the Tlalpan 2020 Cohort. The sample size was estimated considering the associations between hypertension incidence and high sodium intake (HR = 1.25) as well as sleep disorders (HR = 1.58), resulting in a sample size of 2643 participants. These estimates were based on the lowest probability of hypertension events by age group over a 10-year follow-up period. Taking into account the sample size, a two-sided alpha coefficient of 0.05, 80% statistical power, and a 30% attrition rate, the total number of participants required for recruitment was 3436 [10].

This study is a post hoc analysis of the Tlalpan 2020 Cohort, in which we included 1520 participants, of whom 1338 did not develop hypertension (88%), and 182 did develop hypertension (12%). The follow-up time was 29.7 ± 9.6 months for those who did not develop hypertension and 31.9 ± 14.4 months for those who did (*p* = 0.442). The incidence rate of hypertension was 13.6%, with an annual cumulative incidence of 0.45%. Participants were primarily residents of the southern region of Mexico City.

Participants who developed hypertension had higher values in anthropometric measurements, laboratory data, and MOS-SS domains and indices at their initial visit compared to those who did not develop hypertension. Specifically, those who developed hypertension were older (41.8 ± 7.3 years vs. 37.8 ± 9.1 years; *p* < 0.001) and had higher values in body weight (76.8 ± 15.6 kg vs. 70.3 ± 14.4 kg; *p* < 0.001), body mass index (29.3 ± 5.2 vs. 26.7 ± 4.6; *p* < 0.001), waist circumference (95.8 ± 13.8 cm vs. 89.4 ± 12.1 cm; *p* < 0.001), systolic blood pressure (115.1 ± 12.5 mmHg vs. 106.2 ± 10.9 mmHg; *p* < 0.001), and diastolic blood pressure (77.7 ± 8.8 mmHg vs. 71.5 ± 8.4 mmHg; *p* < 0.001). Biochemical markers also showed significant differences, with higher levels of LDL-cholesterol (125.9 ± 28.3 mg/dL vs. 122.0 ± 31.6 mg/dL; *p* = 0.019), glucose (95.3 ± 11.3 mg/dL vs. 92.1 ± 9.0 mg/dL; *p* < 0.001), triglycerides (184.7 ± 102.0 mg/dL vs. 148.4 ± 98.9 mg/dL; *p* < 0.001), and atherogenic index (2.9 ± 0.9 vs. 2.6 ± 0.9; *p* < 0.001) as well as lower levels of plasma iron (92.8 ± 38.7 μg/dL vs. 107.5 ± 42.1 μg/dL; *p* = 0.004) and HDL-cholesterol (45.7 ± 12.3 mg/dL vs. 49.1 ± 12.9 mg/dL; *p* < 0.001) (Table 1).

**Figure 1:**
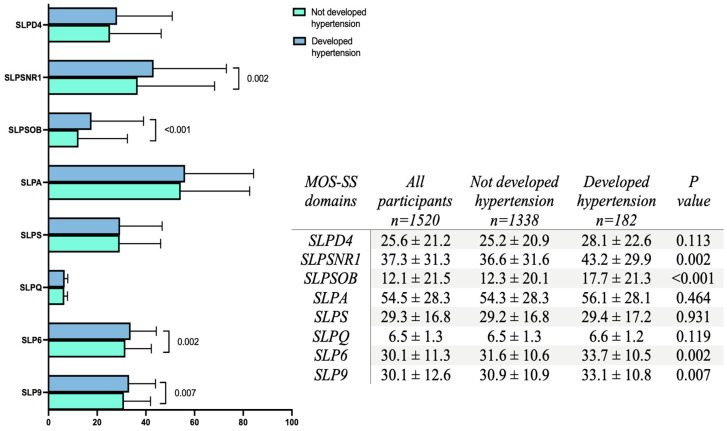
Distribution of MOS-SS scale domains. The bars represent the percentage of each domain assessed by the MOS-SS questionnaire. Blue bars indicate values for participants who developed hypertension, while green bars show values for those who did not. Each bar illustrates the mean ± standard deviation. Group differences were assessed using the unpaired Mann–Whitney U test, with statistical significance set at *p* < 0.05. The domains include SLPD4 (sleep disturbance), SLPSNR1 (snoring), SLPSOB (sleep awakening short of breath or with headache), SLPA (sleep adequacy), SLPS (somnolence), SLPQ (quantity of sleep), SLP6 (short version of sleep problems index), and SLP9 (sleep problems index).

Some MOS-SS domains and indices also showed higher values in participants who developed hypertension: snoring (43.2 ± 29.9 vs. 36.6 ± 31.6; *p* = 0.002), sleep awakening short of breath or with headache (17.7 ± 21.3 vs. 12.3 ± 20.1; *p* < 0.001), short version of sleep problems index (33.7 ± 10.5 vs. 31.6 ± 10.6; *p* = 0.002), and sleep problems index (33.1 ± 10.8 vs. 30.9 ± 10.9; *p* = 0.007) (Figure 1).

The percentage of the sleep problems index for the development of hypertension was 50%, with an odds ratio of 1.5 (95% CI: 1.087 to 2.069). Among those who did not develop hypertension, 31.8% showed an SLP9 > 33.06, while 41.2% of those who developed hypertension had an SLP9 > 33.06 (*p* = 0.014).

In a multiple logistic regression analysis, adjusted for sex, age, body mass index, systolic blood pressure, diastolic blood pressure, LDL-C, HDL-C, and glucose, SLP9 was associated with a higher risk of developing hypertension, with an area under the ROC curve (AUROC) of 0.782 (95% CI: 0.746 to 0.817; *p* < 0.001) and an odds ratio of 1.091 (95% CI: 1.020 to 1.169) (Table 2).

The cut-off point for sleep problems index was ≥33.06 (Youden index = 0.539), with an AUROC of 0.752 (95% CI: 0.669 to 0.834; *p* < 0.001), a sensitivity of 53.97% (95% CI: 41.79 to 65.69), and a specificity of 99.62% (95% CI: 98.89 to 99.90) and a positive likelihood ratio of 142.5. There were no significant differences in the survival analysis (hazard ratio = 0.737, 95% CI: 0.449 to 1.21; chi-square: 1.535; *p* = 0.215).

### 3.2. Analysis of MOS-SS before and after Developing Hypertension

**Figure 2:**
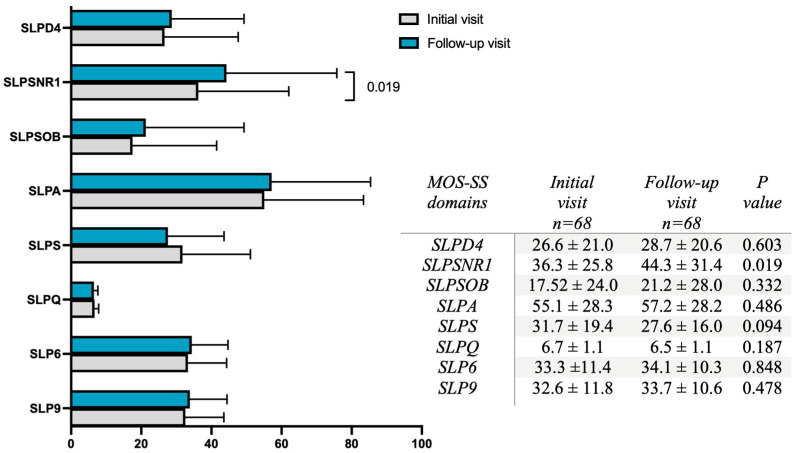
Distribution of the MOS-SS domains. The bars illustrate the percentage of each domain assessed by the MOS-SS scale. Blue bars correspond to values from the follow-up visit, while grey bars represent values from the initial visit. Each bar shows the mean ± standard deviation. Group differences were assessed using the Wilcoxon signed-rank test, with statistical significance set at *p* < 0.05. The domains assessed include SLPD4 (sleep disturbance), SLPSNR1 (snoring), SLPSOB (sleep awakening short of breath or with headache), SLPA (sleep adequacy), SLPS (somnolence), SLPQ (quantity of sleep), SLP6 (sleep problems index 6), and SLP9 (sleep problems index 9).

No significant differences were observed in the sleep problem indices SLP6 and SLP9 between the initial and follow-up visit. However, a significant difference was found in one of the MOS-SS domains: The follow-up visit showed higher percentages of SLPSNR1 (snoring) compared to the initial visit (43.3 ± 31.4 vs. 36.3 ± 25.8; *p* = 0.019) (Figure 2).

## 4. Discussion

Our study revealed a significant association between subjective sleep quality perception and subsequent increases in blood pressure. According to the 2022 National Health Survey (ENSANUT), the prevalence of hypertension among Mexican adults is 27.7% in women and 31.3% in men. Notably, the prevalence is significantly higher in obese adults compared to their non-obese counterparts (58.8% vs. 30.3%) [4]. This finding is consistent with our results, which show that participants with a higher BMI were more likely to develop hypertension.

Emerging evidence highlights the importance of sleep as a crucial component of cardiovascular health. In 2022, the AHA recognized sleep as a fundamental component of cardiovascular health [15]. The assessment of sleep quality encompasses a range of subjective and objective measures, and findings may vary based on methodology, demographics, and other factors. For example, men over the age of 45 who sleep less than six hours per night have a higher risk of hypertension, while women of the same age are at risk if they sleep less than eight hours [16]. Contrarily, a longitudinal study of Mexican adolescents did not find a significant relationship between bedtime and the incidence of hypertension over 14 months, suggesting the need for age-specific study designs [17].

Despite these discrepancies, there is substantial evidence linking poor sleep quality and short sleep duration with the development of hypertension. In adults over 40, late sleep onset and insufficient sleep duration are associated with cardiometabolic health issues [18]. Our study did not find significant differences in sleep duration between participants who developed hypertension and those who did not; however, older participants were more likely to develop hypertension. This indicates that age is a critical but not the sole determinant for the development of hypertension. In pre-menopausal women, factors such as sleep duration, inconsistent sleep patterns, sleep debt, frequent naps, insomnia symptoms, and poor sleep quality were associated with a higher incidence of hypertension [19]. Generally, high scores on self-reported sleep questionnaires correlate with a higher likelihood of hypertension [9]. In our study, participants with higher levels of snoring (SLPSNR1), sleep-related breathing problems (SLPSOB), and sleep problems indices (SLP6 and SLP9) developed hypertension. Longitudinal studies provide robust evidence of the association between sleep problems and hypertension risk. For example, a 3.31-year follow-up study revealed that individuals with good sleep patterns had a 20% lower risk of developing hypertension, with a clear dose–response association between sleep duration and hypertension incidence [1]. In our study population, with an average follow-up of 30.8 months, the incidence of hypertension was 13.6%, with an annual cumulative incidence of 0.45. According to our analysis, having an SLP9 index value >33.06 confers a high risk of developing hypertension, and once the population was adjusted for sex, age, BMI, blood pressure, lipids, and glucose, the risk of developing hypertension due to a high SLP9 index remained.

Obstructive sleep apnea (OSA), the most common form of sleep-disordered breathing, frequently occurs alongside hypertension. Its pathophysiology involves sympathetic hyperactivity, inflammation, pharyngeal collapse, intravascular fluid retention, nocturnal energy expenditure, and metabolic disturbances, with a dose–response relationship between OSA severity and hypertension severity [20]. Sleep-disordered breathing is a common but often underdiagnosed condition affecting approximately 17% of middle-aged women and 34% of middle-aged men [21]. Epidemiological studies have linked sleep-disordered breathing to an increased risk of metabolic diseases, including hypertension [22]. In our matched analysis of the follow-up of subjects who developed hypertension, SLPSNR1 (self-reported snoring) showed significant differences, highlighting its role in the development of hypertension. Interestingly, in a Japanese cohort, sleep-disordered breathing was associated with a high prevalence of hypertension even among non-obese subjects, highlighting the clinical impact of sleep-disordered breathing on the development of hypertension [23].

Poor self-reported sleep quality in our study was associated with the development of hypertension, with sleep-related breathing problems emerging as the primary concern. A limitation of our study is the relatively small number of participants who both developed hypertension and completed the MOS-SS questionnaire at follow-up visits, as the original project did not specifically focus on sleep. Nonetheless, the analyses we conducted provide significant findings that emphasize the importance of early diagnosis and treatment of sleep-disordered breathing.

In conclusion, subjective sleep quality and self-reported sleep-disordered breathing are associated with the incidence of hypertension in a middle-aged population. Additionally, hypertension is associated with snoring problems in this demographic. These findings highlight the importance of addressing sleep health as a crucial target for the prevention and treatment of hypertension.

## Figures and Tables

**Table 1:** Clinical characteristics and laboratory data of the study population.

	All Participants *n* = 1520	Not Developed Hypertension *n* = 1338	Developed Hypertension *n* = 182	*p*-Value
Sex (Woman), *n* (%)	968 (63.6)	858 (64.1)	110 (60.8)	0.414
Age (years), mean ± SD	38.3 ± 8.9	37.8 ± 9.11	41.8 ± 7.3	<0.001
Weigth (kg), mean ± SD	71.3 ± 14.6	70.3 ± 14.4	76.8 ± 15.6	<0.001
Height (m), mean ± SD	1.62 ± 0.1	1.62 ± 0.1	1.61 ± 0.1	0.881
BMI (kg/m^2^), mean ± SD	27.1 ± 4.7	26.7 ± 4.6	29.3 ± 5.2	<0.001
Waist circumference, mean ± SD	90.3 ± 12.3	89.4 ± 12.1	95.8 ± 13.8	<0.001
Systolic blood pressure (mm Hg), mean ± SD	108.8 ± 11.1	106.2 ± 10.9	115.1 ± 12.5	<0.001
Diastolic blood pressure (mm Hg), mean ± SD	72.9 ± 8.7	71.5 ± 8.4	77.7 ± 8.8	<0.001
Heart rate (beats/minute), mean ± SD	65.4 ± 8.2	65.2 ± 8.1	66.6 ± 8.9	0.011
SLP9 > 33.06, *n* (%)	608 (40)	521 (38.9)	87 (47.8)	0.024
Uric acid (2.4–7.0 mg/dL), mean ± SD	5.3 ± 1.3	5.3 ± 1.3	5.4 ± 1.4	0.229
Creatinine (0.5–1.2 mg/dL), mean ± SD	0.79 ± 0.2	0.8 ± 0.1	0.8 ± 0.2	0.597
Total cholesterol (<200 mg/dL), mean ± SD	189 ± 35.6	188.1 ± 36.1	191.7 ± 39.9	0.228
HDL-C (≥60 mg/dL), mean ± SD	48.7 ± 12.8	49.1 ± 12.9	45.7 ± 12.3	<0.001
LDL-C (<100 mg/dL), mean ± SD	122.6 ± 31.1	122.0 ± 31.6	125.9 ± 28.3	0.019
Glucose (74–106 mg/dL), mean ± SD	92.6 ± 8.8	92.1 ± 9.0	95.3 ± 11.3	<0.001
Triglycerides (<150 mg/dL), mean ± SD	153.8 ± 100.5	148.4 ± 98.9	184.7 ± 102	<0.001
Atherogenic index (<4.5), mean ± SD	2.7 ± 0.9	2.6 ± 0.9	2.9 ± 0.9	<0.001
Sodium (136–145 mmoL/L), mean ± SD	138.1 ± 2.5	137.9 ± 6.5	136.3 ± 14.8	0.255
Sodium in urine (40–220 mmoL/24 h), mean ± SD	130.8 ± 57.6	130.4 ± 57.3	131.0 ± 59.8	0.934
Urine volume (<2000 mL/24 h), mean ± SD	1553 ± 73.4	1546 ± 71.1	1585 ± 72.5	0.512
Plasma iron (33–193 μg/dL), mean ± SD	105.8 ± 41.8	107.5 ± 42.1	92.8 ± 38.7	0.004

Qualitative variables are presented as percentages, while quantitative variables are presented as means and standard deviations. Group differences were evaluated using Fisher’s exact test and Mann–Whitney test, respectively. A *p*-value < 0.05 was considered indicative of statistically significant differences. Abbreviations: BMI (body mass index); HDL-C (high-density lipoprotein); LDL-C (low-density lipoprotein).

**Table 2:** Multivariate Logistic Regression Analysis of Factors Associated with the Development of hypertension.

	Odds Ratio	95% CI	*p*-Value
Sex	1.109	0.744 to 1.656	0.613
Age	1.051	1.027 to 1.077	<0.001
BMI	1.068	1.025 to 1.112	0.001
SBP	1.038	1.009 to 1.053	0.005
DBP	1.057	1.027 to 1.088	<0.001
HDL-C	0.989	0.989 to 1.005	0.188
LDL-C	0.997	0.991 to 1.003	0.402
Glucose	1.005	0.984 to 1.026	0.628
SLPD4	0.961	0.939 to 0.984	0.001
SLPSNR1	0.996	0.990 to 1.003	0.273
SLPSOB	0.998	0.988 to 1.007	0.693
SLPA	0.998	0.991 to 1.006	0.677
SLPS	0.985	0.971 to 0.999	0.048
SLPQ	1.114	0.957 to 1.296	0.161
SLP6	1.006	0.964 to 1.049	0.078
SLP9	1.091	1.020 to 1.169	0.011

Multiple logistic regression analysis with hypertension as the dependent variable and adjusted for age, sex, BMI, SBP, DBP, HDL-C, LDL-C, and glucose was performed to determine the risk conferred by the MOS-SS scale domains and indices. Abbreviations: BMI (body mass index), SBP (systolic blood pressure), DBP (diastolic blood pressure), HDL-C (high-density lipoprotein), LDL-C (low-density lipoprotein), SLPD4 (sleep disturbance), SLPSNR1 (snoring), SLPSOB (sleep awakening short of breath or with headache), SLPA (sleep adequacy), SLPS (somnolence), SLPQ (quantity of sleep), SLP6 (sleep problems index 6), and SLP9 (sleep problems index 9).

## Data Availability

The data presented in this study are available on request from the corresponding author. The data are not publicly available due to restrictions associated with the protection of personal data in force in Mexico.

## Supplementary Materials

The following supporting information can be downloaded at: https://www.mdpi.com/article/10.3390/jcm13206089/s1.
